# Human fetal mesenchymal stem cell secretome enhances bone consolidation in distraction osteogenesis

**DOI:** 10.1186/s13287-016-0392-2

**Published:** 2016-09-10

**Authors:** Jia Xu, Bin Wang, Yuxin Sun, Tianyi Wu, Yang Liu, Jinfang Zhang, Wayne Yukwai Lee, Xiaohua Pan, Yimin Chai, Gang Li

**Affiliations:** 1 Department of Orthopaedic Surgery, Shanghai Jiao Tong University Affiliated Sixth People’s Hospital, Shanghai, People’s Republic of China; 2 Department of Orthopaedics & Traumatology, Stem Cells and Regeneration Laboratory, Li Ka Shing Institute of Health Sciences, Faculty of Medicine, The Chinese University of Hong Kong, Prince of Wales Hospital, Shatin, Hong Kong, SAR People’s Republic of China; 3 The CUHK-ACC Space Medicine Centre, The Chinese University of Hong Kong Shenzhen Research Institute, Shenzhen, People’s Republic of China; 4 Department of Orthopaedics and Traumatology, Bao-An People’s Hospital, Shenzhen, People’s Republic of China

**Keywords:** hFMSCs, Secretome, Osteogenesis, Distraction osteogenesis, Bone consolidation

## Abstract

**Background:**

Distraction osteogenesis (DO) is one of the most dramatic reconstructive techniques for inducing bone regeneration, but it involves an undesirably long period for bone consolidation. Developing innovative approaches to enhance bone consolidation is a burning need. Human fetal mesenchymal stem cells (hFMSCs) have been shown to express more primitive developmental genes than those of human adult mesenchymal stem cells (hAMSCs), which is a preferable source for cell therapy and tissue regeneration. In the present study, we investigated the immunogenicity of using the human mesenchymal stem cell (MSC) secretome on rat cells, the effects of secretome on osteogenic differentiation of rat bone marrow-derived MSCs (rBMSCs), and the potential application of hFMSC secretome in promoting bone consolidation in a rat DO model.

**Methods:**

Secretome was collected from MSC culture and was used to treat rBMSCs. Following secretome treatment, cell proliferation, alkaline phosphatase staining, Alizarin Red S staining, and mRNA expression of osteogenic differentiation-related genes (including ALP, Runx2, OCN, OPN, and Osx) in the rBMSCs were checked, as well as mixed rat peripheral blood lymphocyte reaction. hFMSC secretome was injected locally into the regenerates from the end of lengthening every 3 days in the rat DO model, until termination. The regenerates were subject to weekly x-rays, micro-computed tomography (μCT) and mechanical testing examination. The bone quality was assessed by histology and immunohistochemistry examinations.

**Results:**

Compared to the secretome from rBMSCs and hAMSCs, hFMSC secretome had the best osteogenic induction ability and low immunogenicity. hFMSC secretome with different doses showed no effect on cell viability. hFMSC secretome at the dose of 100 μg/μl could significantly increase the expression of alkaline phosphatase and all the osteogenic marker genes, as well as the amount of calcium deposits in the rBMSCs. Finally, the local application of hFMSC secretome in distraction regenerates in a rat DO model significantly improved bone consolidation according to the results of μCT, mechanical test, and histological and immunohistochemistry analysis.

**Conclusions:**

The current study demonstrated that hFMSC secretome promotes osteogenesis of rBMSCs and bone consolidation during DO. hFMSC secretome may be a new therapeutic strategy to enhance bone consolidation in patients undergoing DO treatment.

## Background

Distraction osteogenesis (DO) is a dramatic reconstructive technique that promotes bone regeneration by applying controlled gradual traction between the osteotomy cuts. The DO procedure that was first described by Ilizarov [1], with specific rate and rhythm, could correct a variety of orthopedic deformities and malformations with remarkable results [2]. “Tension-stress principle” is the biological basis for regenerating large segments of bone that have been lost due to congenital deformity, trauma, or chronic osteomyelitis [2, 3]. Nevertheless, despite its widespread application in the clinic, patients undergoing DO procedure with bulk external fixator must endure an undesirably long treatment period to allow bone consolidation. Therefore, innovative approaches to enhance bone consolidation are in burning need [4].

Various adult mesenchymal stem cells (MSCs) and their derivatives have been transplanted into the damaged area to promote tissue repair in both humans and animals [5, 6]. Because of the poor differentiation and survival rates after MSC transplantation, the engrafted MSCs promote tissue regeneration mainly through paracrine effects [7, 8]. Serum-free conditioned medium derived from human adult MSCs (hAMSCs) was applied to accelerate bone formation in preclinical animal models [9]. Moreover, human fetal MSCs (hFMSCs) have been demonstrated recently to have growth promoting potential; express more primitive developmental genes, which are preferable for cell therapy and tissue regeneration [10–12]. hFMSCs have superior cell proliferation capacity, more robust osteogenic potential, and lower immunogenicity, compared to hAMSCs [13].

The spectrum of regulatory and trophic factors secreted by stem cells including growth factors, cytokines, exosomes, and microRNAs is broadly defined as the secretome [14]. Stem cells, such as MSCs, are attracted to the damaged tissue site where they produce the secretome that enhances angiogenesis, reduces inflammation, promotes tissue repair, and inhibits fibrosis and cell apoptosis [15–17]. Application of cell-free secretome could avoid the limitations associated with cell therapy, such as immune incompatibility, longer waiting time, and higher costs for cell preparation [18, 19].

In this study, we introduce a method of generating secretome from MSCs and investigate their effects on osteogenic differentiation of rat bone marrow-derived MSCs (rBMSCs) and the effects of local administration of hFMSC secretome on bone consolidation in a rat DO model. As an alternative to cell therapy, we hypothesize that such cell-free secretome from hFMSCs may have the similar regenerative potential to enhance bone consolidation in patients undergoing DO treatment.

## Methods

### Cell culture and secretome preparation

The bone marrow MSCs were isolated as previously described [19, 20]. Briefly, cells were cultured in modified Eagle’s medium of Alpha (α-MEM; Invitrogen, USA) supplemented with 10 % fetal bovine serum (FBS; Gibco, USA) and 1 % penicillin/streptomycin (Gibco, USA) at 37 °C with 5 % CO_2_ and 95 % humidity. The MSCs from passages 3–6 were used in the experiments. Meanwhile, when MSCs reached 80 % confluence they were placed in serum-free α-MEM, which was used as the positive control in the animal studies, and incubated for 24 h in 5 % CO_2_ in a humidified condition, after which the conditioned medium was collected and centrifuged to purify for 10 min at 4 °C, 4000 g. Then 15 ml conditioned medium was re-centrifuged with Amicon Ultra Centrifugal Filters (Millipore, USA) for 60 min at 4 °C, 4000 g. Around 300–400 μl supernatant solution could be collected as cell-free secretome each time. For in vitro experiments, 100 μl secretome was added into 3 ml osteogenic induction medium (OIM), while for in vivo studies 100 μl secretome was locally injected to the regenerate zone. The protein content was measured using the BCSA kit (Thermo Scientific, Rockford, IL, USA) according to the manufacturer’s instruction. The concentrations of secretome used in vitro and vivo were 100 μg/μl and 3 mg/μl, respectively.

### Cell viability assay

The rBMSCs were trypsinized and placed in a flat-bottomed 96-well plate at an initial density of 5000 cells per well. After 24 h of incubation, the medium was changed to hFMSC secretome containing medium at different doses (0, 10 μg/μl, 25 μg/μl, 50 μg/μl, 100 μg/μl, 200 μg/μl). Cells were incubated at 37 °C for 48 and 72 h. The proliferation was determined by methyl thiazolyl tetrazolium (MTT) reduction assay. After incubation, rBMSCs were treated using the MTT solution with a final concentration of 0.5 mg/ml for 4 h at 37 °C. The dark blue formazan crystals formed in intact cells were solubilized with 150 μl DMSO and the plate was shaken for 10 min. The absorbance was measured at 570 nm with a microplate reader.

### Osteogenic differentiation of rBMSCs

Osteogenic differentiation induction was performed as previously described [21]. Briefly, the rBMSCs were trypsinized and placed in a 12-well plate at an initial density of 5000 cells/cm^2^. When over 80 % confluence was reached, the medium was replaced with osteogenic induction medium (OIM) including 1 nM dexamethasone, 50 μM L-ascorbic acid-2-phosphate and 20 mM β*-*glycerophosphate. The osteogenic differentiation was evaluated by alkaline phosphatase (ALP) staining at day 3, Alizarin Red S staining at days 7 and 14, and quantitative real-time PCR examination of various osteogenic marker genes at days 3 and 10. Triplicate tests were conducted in each experiment.

### ALP staining

After rBMSCs were treated with different kinds of secretome from rBMSCs, hFMSCs, and hAMSCs, as well as different doses of hFMSC secretome (0, 10 μg/μl, 25 μg/μl, 50 μg/μl, 100 μg/μl, and 200 μg/μl) for 3 days, the cells were washed with phosphate-buffered solution (PBS) twice and fixed with 70 % ethanol for 10 min. The cells were equilibrated by ALP buffer (0.1 M NaCl, 0.1 M Tris-HCl, 50 mM MgCl_2_.6H_2_O, pH 9.5) for 5 min twice, and incubated with ALP substrate solution (5 μl BCIP and 10 μl NBT in l ml ALP buffer) at 37 °C in the dark for 60 min, after which the reaction was stopped with distilled water and the plate was dried before taking photos.

### Alizarin Red S staining

Alizarin Red S staining was performed to evaluate calcium deposit formation. After 7 days of osteogenic induction with different kinds of secretome (100 μg/μl) from rBMSCs, hFMSCs, and hAMSCs, and 7 and 14 days with different doses of hFMSC secretome (0, 10 μg/μl, 25 μg/μl, 50 μg/μl, 100 μg/μl, and 200 μg/μl), rBMSCs were washed with PBS and fixed with 70 % ethanol for 10 min, then the cells were stained with Alizarin Red S (pH 4.2) for 10 min at room temperature and washed three times with distilled water. To qualify the mineralization, the monolayer was eluted with 10 % cetylpyridinium chloride (CPC; Sigma), and the absorbance was measured at 570 nm.

### RNA extraction and quantitative real-time PCR

After rBMSCs were treated with hFMSC secretome at a dose of 100 μg/μl for 3 and 10 days, total cellular RNA was extracted with RNA Mini Kit (Invitrogen), and reversely transcribed into cDNA with M-MLV reverse transcriptase (Invitrogen) according to the manufacturer’s instructions. Real-time PCR was performed using the Step One Plus Real-Time PCR System (Applied Biosystems, USA). The reaction conditions consisted of 10 μl reaction volumes with diluted cDNA template 1 μl, 5 μl SYBR-Green Master Mix (2×), 3.4 μl PCR-grade water, and 0.6 μl of each primer (10 μM). The amplification procedure was carried out as follows: first at 95 °C for 5 min, and then 40 cycles of 95 °C for 15 s and 60 °C for 60 s. Primer sequences were as follows: glyceraldehyde-3-phosphate dehydrogenase (GAPDH) forward: 5′GGCATGGACTGTGGTCATGAG3′, reverse: 5′TGCACCACCAACTGTTAGC3′; ALP forward: 5′ACCATTCCCACGTCTTCACATTT3′, reverse: 5′AGACATTCTCTCGTTCACCGCC3′; Runt-related transcription factor 2 (Runx2) forward: 5′ACTTCCTGTGCTCGGTGCT3′, reverse: 5′GACGGTTATGGTCAAGGTGAA3′; osteocalcin (OCN) forward: 5′CCTCACACTCCTCGCCCTATT3′, reverse: 5′CCCTCCTGCTTGGACACAAA3′; osteopontin (OPN) forward: 5′GTACCCTGATGCTACAGACG3′, reverse: 5′TTCATAACTGTCCTTCCCAC3′; Osterix (Osx) forward: 5′CCAGGCAACACTCCTACTCC3′, reverse: 5′GCCTTGCCATACACCTTGC3′. The relative quantification of gene expression was analyzed with the values of 2^–ΔΔCT^, normalized with GAPDH expression level.

### Mixed rat peripheral blood lymphocyte reaction

For the mixed rat peripheral blood lymphocyte reaction, 1 × 10^5^ peripheral blood lymphocytes (rPBLs) isolated from healthy rats were added into 100 μl α-MEM and plated on each well in 96-well plates. After 4 h of culture, different doses (0, 50 μg/μl, 100 μg/μl, and 200 μg/μl) of hFMSC secretome and hAMSC secetome in 100 μl α-MEM were added into each well. rPBSL culture with serum-free α-MEM served as the baseline control. After an additional 1, 3, and 5 days of culture, the proliferation of rPBLs was determined by bromodeoxyuridine (BrdU) incorporation assay according to the manufacturer’s manual (Cell Signaling Technology, USA). Optical density was measured at 450 nm.

### Animal surgery and distraction osteogenesis protocol

Twenty-four 12-week-old SD male rats were used in our study. Before surgery, each rat was anesthetized with a solution of 0.2 % (vol/vol) xylazine and 1 % (vol/vol) ketamine in PBS. All animals were subjected to a right tibia transverse osteotomy procedure with a closed fracture at the midshaft near the fibula-tibia junction under sterile condition. Of note, the periosteum of the tibia should be retained as much as possible. A monolateral external distraction fixator (Tianjing Xinzhong Company, China) was placed to fix proximal and distal segments of the osteotomy site. Surgical incisions were then sutured sequentially. All rats were randomized equally into three groups with the following treatments: PBS group (*n* = 8); medium group (*n* = 8) and secretome group (*n* = 8).

The distraction protocol consisted of three phases: a latency phase of 5 days; a 10-day active lengthening phase (1 mm/day, in two steps, every 12 h); and a consolidation phase of 6 weeks. From the beginning of the consolidation phase, three groups received injection of PBS (100 μl), serum-free α-MEM (100 μl), and secretome (100 μl), respectively, into the distraction gap every 3 days until termination. All rats received subcutaneous injection of calcein (10 mg/kg; Sigma-Aldrich, St. Louis, MO, USA) at the beginning of the consolidation phase, and xylenol orange (30 mg/kg, Sigma-Aldrich) 3 days before termination (day 57 after surgery). Bilateral tibias were harvested, strapped free of muscle, and processed for further examinations.

### Digital radiographs

At the end of lengthening, a weekly anterior-posterior x-ray including the distraction zone was taken until termination using a digital x-ray machine (MX-20, Faxitron X-Ray Corp., Wheeling, IL, USA) under an exposure time of 6000 ms and a voltage of 32 kv.

### Micro-computed tomography (μCT)

The structural change within the distraction zone in the rat DO model was quantitatively assessed with μCT as previously described [22]. Briefly, all the specimens were imaged using a high-solution μCT (μCT40, Scanco Medical, Bassersdorf, Switzerland) at a custom isotropic resolution of 8 μm isometric voxel size with a voltage of 70 kV and a current of 114 μA. Three dimensional (3D) reconstructions of the mineralized callus were performed using a global threshold (165 mg hydroxyapatite/cm^3^), and a Gaussian filter (sigma = 0.8, support = 2) was applied to suppress noise. Sagittal images of the distraction zone were used to perform 3D histomorphometric analysis. The region of interest was defined as the distraction zone (regenerate) between the two closest proximal and distal half-pins. Low- and high-density mineralized tissues were reconstructed using different thresholds (low attenuation = 158, high attenuation = 211) using our established evaluation protocol with a small modification [23]. We selected the volume of interest to cover the distraction zone. The high-density tissues represented the newly formed highly mineralized bone, while the low ones represented the newly formed callus. Bone volume/total tissue volume (BV/TV) of each specimen was recorded for analysis.

### Four-point bending mechanical testing

A mechanical test was performed within 24 h after termination at room temperature. The contralateral tibia was tested as an internal control. A four-point bending device (H25KS; Hounsfield Test Equipment Ltd., UK) with a 250 N load cell was used to test the tibia to failure. The tibias were loaded in the anterior-posterior direction with the inner and outer span of the blades set as 8 and 18 mm, respectively. The long axis of the tibia was placed perpendicular to the blades during the test [23]. The modulus of elasticity (E-modulus), ultimate load, and energy to failure were obtained and analyzed with built-in software (QMAT Professional; Tinius Olsen, Inc., Horsham, PA, USA). The biomechanical properties of the new bone were expressed as percentages of the contralateral intact bone properties.

### Histology and immunohistochemistry

All tibias were initially fixed in 10 % formalin for 48 h. Half of them were followed by decalcification in 10 % EDTA solution for 3 weeks and embedded into paraffin. Thin sections (5 μm) were cut by a rotary microtome (HM 355S, Thermo Fisher Scientific, Inc., Germany) along the long axis of each tibia in the sagittal plane. After deparaffinization, immunohistochemistry staining was performed. The other half of the specimens were managed by gradient alcohol dehydration, xylene defatting, and undecalcification embedded in methyl methacrylate. Thin (5 μm) and thick (10 μm) sections were cut with the RM2155 hard tissue microtome (Leica, Wetzlar, Germany) along the long axis of the tibia, respectively. The 5-μm sections were stained with Trichrome Goldner and Von Kossa for static histomorphometric analysis, while the unstained 10-μm ones were used for dynamic histomorphometric measurements, which contained singled labeled surface (sL.S), double-labeled surface (dL.S), ratio of mineralizing surface to bone surface (MS/BS, calculated as double plus half of single-labeled surfaces (sL.S)), mineral apposition rate (MAR), bone formation rate per unit of bone surface (BFR/BS), bone formation rate of bone volume (BFR/BV), and bone formation rate of tissue volume (BFR/TV).

Immunohistochemistry staining was performed using a standard protocol as previously reported [24]. Secretions were treated with primary antibodies to rabbit osterix (Osx; Abcam, 1:100, ab22552) and osteocalcin (OCN; Santa Cruz, 1:100, sc30045) overnight at 4 °C; a horseradish peroxidase-streptavidin detection system (Dako, USA) was used, followed by counterstaining with hematoxylin. The positive stained cell numbers in the whole distraction zone per specimen in three sequential sections (50 μm, 150 μm, and 250 μm) per rat in each group were counted and compared, and were expressed as the percentages of the bone volume.

### Statistical analysis

All quantitative data were analyzed using SPSS 18.0 software for windows (SPSS, Chicago, IL, USA). Non-parametric test was used for comparison of mean values with *p* < 0.05 considered as statistically significant.

## Results

### Effect of different kinds of secretome on osteogenic differentiation of rBMSCs

**Fig. 1:**
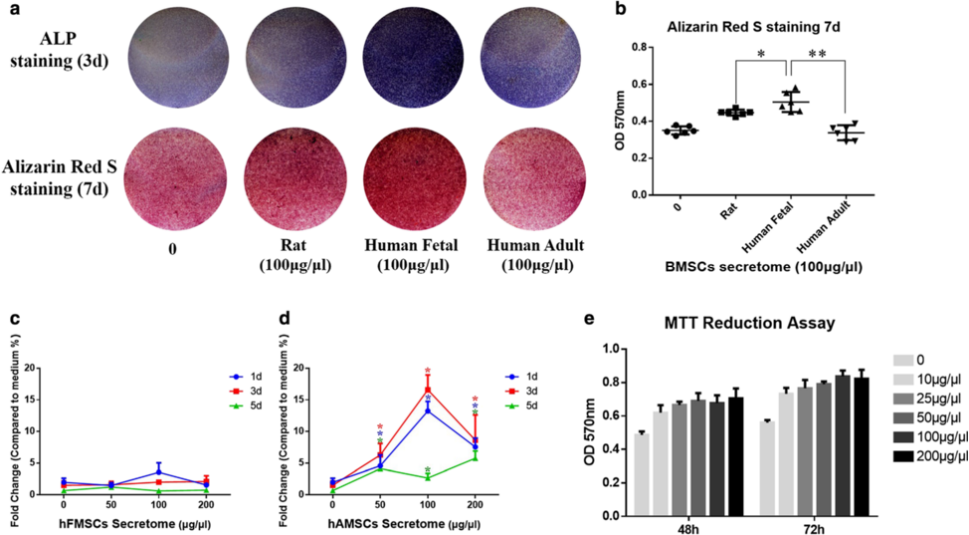
Secretome from human fetal mesenchymal stem cells (*hFMSCs*) had more osteogenic potential and lower immunogenicity than that from rat bone marrow-derived mesenchymal stem cells (*r* *BMSCs*) and human adult mesenchymal stem cells (*hAMSCs*), as well as no effect on cell viability. **a** Both the alkaline phosphatase (*ALP*) staining and Alizarin Red S staining showed that more calcium deposits could be found after hFMSC secretome treatment. **b** The semi-quantitative results of Alizarin Red S staining demonstrated that significantly more mineralization was seen in the hFMSC secretome group. **c** Immunogenicity was determined by mixed rat peripheral blood lymphocyte reaction. The hFMSC secretome had no significant effect on rat peripheral blood lymphocyte proliferation. **d** Significant lymphocyte proliferation was stimulated after administration of hAMSC secretome. **e** Cell viability was evaluated by the MTT assay. The optical density (*OD*) level of rBMSCs treated with hFMSC secretome showed no significant difference among the doses from 10 μg/μl to 200 μg/μl. *p < 0.05, **p < 0.01. *d* days

To compare the osteogenic effects of rBMSC secretome, hFMSC secretome, and hAMSC secretome on the osteogenic differentiation of rBMSCs, ALP and Alizarin Red S staining were performed. The results showed that both rat and human secretome could induce osteogenic differentiation of rBMSCs. Furthermore, the hFMSC secretome demonstrated the strongest osteogenic induction ability (Fig. 1a, b).

### Immunogenicity of secretome from hFMSCs and hAMSCs

The responses of rat peripheral blood lymphocyte culture treated with hFMSC secretome and hAMSC secretome were tested by mixed lymphocyte reaction. The results showed a dramatic lymphocyte proliferation under hAMSC secretome treatment in a concentration -dependent manner at days 1 and 3. At day 5, the low BrdU incorporation indicated cells might reach the stationary phase (Fig. 1d). In contrast, the hFMSC secretome treatment at all the tested concentrations did not induce significant lymphocyte proliferation (Fig. 1c).

### Different doses of hFMSC secretome had no effect on cell viability but promoted osteogenic differentiation of rBMSCs

**Fig. 2:**
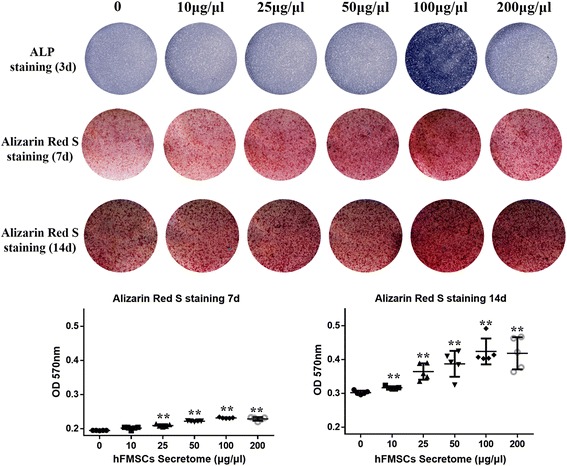
Human fetal mesenchymal stem cell (*hFMSC*) secretome promoted osteogenic differentiation of rBMSCs in vitro. The alkaline phosphatase (*ALP*) staining was conducted after 3 days of treatment with secretome with different doses in OIM, and the Alizarin Red S staining and its quantitated measurement were performed after 7 and 14 days administration of secretome. Significantly more mineralization was seen in the hFMSC secretome group with a dose of 100 μg/μl. **p < 0.01. *d* day, *OD* optical density

**Fig. 3:**
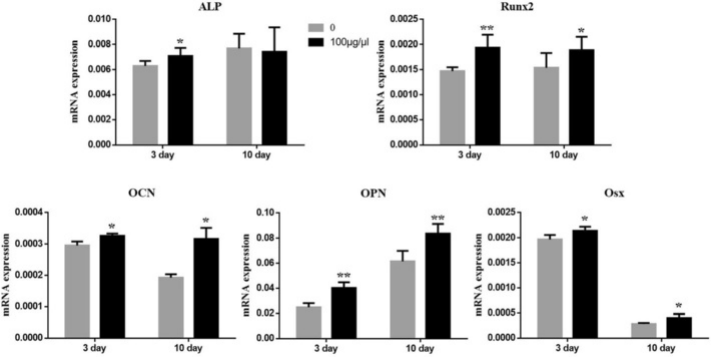
hFMSC secretome upregulated levels of osteogenic mRNA expression in rBMSCs. Osteogenic marker gene expressions were detected by quantitative real-time PCR after treatment with secretome at the dose of 100 μg/μl in OIM for 3 and 10 days. *p < 0.05, **p < 0.01, compared with OIM. *ALP* alkaline phosphatase, *OCN* osteocalcin, *OPN* osteopontin, *Osx* osterix, *Runx2* Runt-related transcription factor 2

To investigate the effect of hFMSC secretome on cell viability, the MTT assay was performed. The results showed that there was no significant difference among the five groups with different doses of secretome (excluding the dose of 0) during 48- and 72-h culture (Fig. 1e). To clarify the effect of different doses of hFMSC secretome on osteogenesis of rBMSCs in vitro, ALP and Alizarin Red S staining were performed at day 3, and days 7 and 14, respectively. The expression of alkaline phosphatase and the amount of calcium deposits were remarkably increased in the group with a dose of 100 μg/μl. The quantitative results showed that hFMSC secretome at a dose of 100 μg/μl could significantly increase calcium nodule formation compared to other doses (Fig. 2). Furthermore, the real time PCR results demonstrated a remarkable increase in the expression of Runx2, OCN, OPN, and Osx in the secretome group with the dose of 100 μg/μl at days 3 and 10. The ALP in the secretome group was significantly upregulated at day 3, but showed no significant difference at day 10 (Fig. 3).

### Radiographic assessment of the distraction zone

**Fig. 4:**
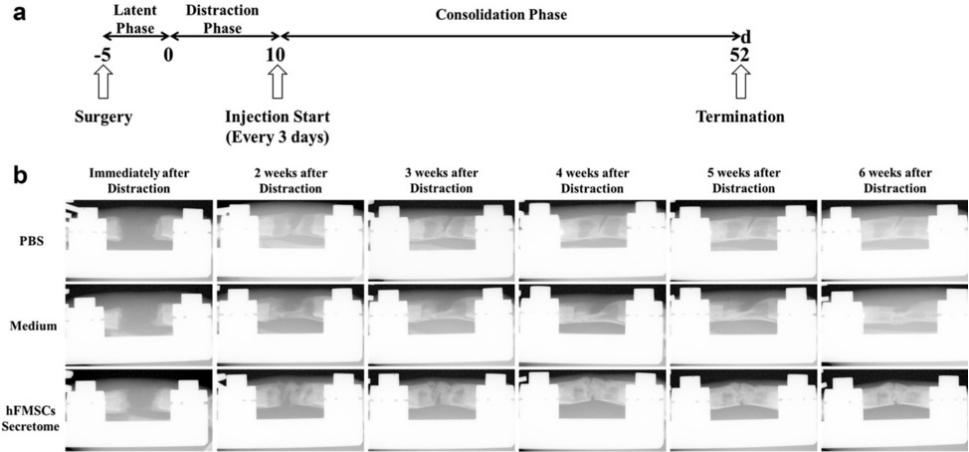
Animal experimental design and representative x-rays of distraction regenerate at various time points. **a** After a 5-day latency period, distraction was initiated over 10 days at 1 mm/day in two equal increments. Local injection of PBS, serum-free α-MEM, and secretome started from the beginning of the consolidation phase, and every 3 days thereafter until termination. **b** Little callus was seen in the gap immediately after distraction in three groups, while more continuous callus appeared in the secretome group as time went on, especially at the end of the consolidation phase, whereas an obvious gap was still seen in the other two groups. *d* day, *hFMSC* human fetal mesenchymal stem cell, *PBS* phosphate-buffered saline

**Fig. 5:**
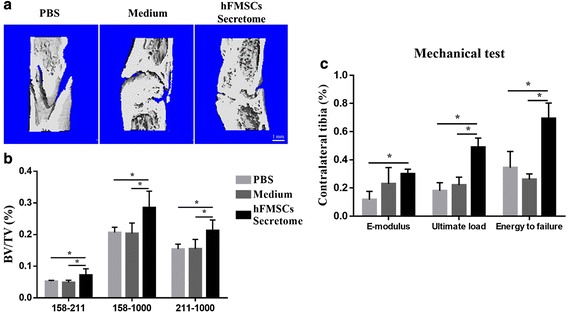
Secretome administration improved the quality of new callus via μCT analysis and mechanical test. **a** 3D μCT images of the tibia distraction zone in three groups confirmed more continuous callus was found after secretome treatment. **b** μCT analysis data showed bone volume/total tissue volume (*BV/TV*) of newly consolidated bone in the secretome group was much higher than that in the phosphate-buffered saline (*PBS*) and medium groups. Attenuation above 158 represented total mineralized tissue, and attenuation between 158 and 211 represented the new callus. **c** Better mechanical properties (including the modulus of elasticity (*E-modulus*), ultimate load, and energy to failure) of distraction tibia were shown in the secretome group (%). *p < 0.05, compared with PBS or medium group, *n* = 8. *hFMSC* human fetal mesenchymal stem cell

Representative series of x-rays across the time-course of DO showed the progression of bone consolidation (Fig. 4). Little callus was observed in the gap at the end of distraction in all groups. However, as time went on, more callus formation was found in the secretome treatment group compared to the medium group and PBS group until termination. A similar result was found in the 6-week images using μCT (Fig. 5a). The value of BV/TV at week 6 indicated that more newly formed mineralized bone was detected in the secretome treatment group compared to the other two groups, while there was no remarkable difference between the medium group and the PBS group (Fig. 5b).

### Mechanical testing

The results of four-point bending mechanical testing in the secretome treatment group showed a significant improvement in ultimate load and energy to failure compared to the other two groups after being normalized with the contralateral intact tibia. However, there was no significant difference between the secretome treatment group and the medium group in E-modulus (Fig. 5c).

### Histological analysis

**Fig. 6:**
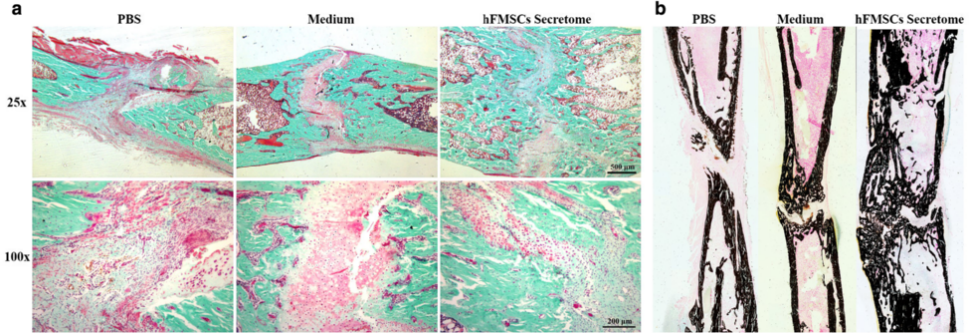
Histological analysis showed that the secretome intervention accelerated new callus consolidation. **a** Representative sections stained with Trichrome Goldner showing better quality callus formation in the secretome group than in the other two groups. **b** Von Kossa staining clearly showed that most of the new bone has been consolidated and the continuity of the cortical bone and bone marrow cavities was evident in the secretome group at week 6. *hFMSC* human fetal mesenchymal stem cell, *PBS* phosphate-buffered saline

**Fig. 7:**
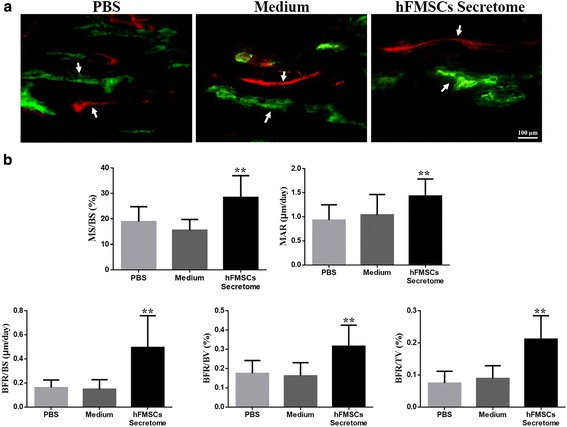
Dynamic histomorphometric measurements showed more quantitative bone formation in the secretome group. **a** *Arrows* point to the calcein and xylenol orange labeling in representative images of three groups. **b** Quantitative measurements of dynamic histomorphometric parameters containing MS/BS, MAR, BFR/BS, BFR/BV, and BFR/TV were significantly increased in the secretome group. **p < 0.01, compared to PBS group and medium group. *BFR/BS* bone formation rate per unit of bone surface, *BFR/BV* bone formation rate of bone volume, *BFR/TV* bone formation rate of tissue volume, *hFMSC* human fetal mesenchymal stem cell, *MAR* mineral apposition rate, *MS/BS* ratio of mineralizing surface to bone surface, *PBS* phosphate-buffered saline

**Fig. 8:**
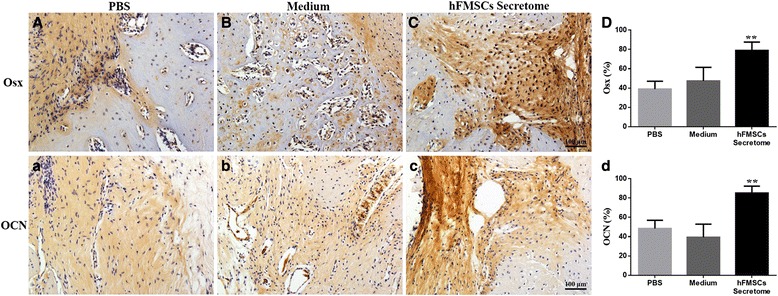
Immunohistochemical analysis of the percentages of Osx- and OCN-positive cells in the distraction zone. The secretome treatment has significantly increased numbers of Osx (**A**–**D**) and OCN (**a**-**d**) positive cells (*brown*) compared to the PBS and medium treatment (**p < 0.01). *hFMSC* human fetal mesenchymal stem cell, *OCN* osteocalcin, *Osx* osterix, *PBS* phosphate-buffered saline

Representative sections from three groups stained with Trichrome Goldner and Von Kossa are shown in Fig. 6. More chondrocytes were detected in the PBS group and the medium treatment group than in the secretome treatment group, suggesting that the newly formed chondrocytes have not been mineralized completely in the two groups. In contrast, most of the new bone had been consolidated and the continuity of the cortical bone and bone marrow cavities were reconstructed in the secretome treatment group. Dynamic histomorphometric data are presented in Fig. 7a and b. MS/BS, MAR, BFR/BS, BFR/BV, and BFR/TV were significantly increased in the secretome treatment group compared to the medium group and the PBS group, suggesting that bone consolidation was enhanced by the secretome treatment. Osx and OCN immunohistochemistry staining confirmed more Osx- and OCN-positive cells in the new bone zone in the secretome treatment group than in the other two groups (Fig. 8).

## Discussion

In the present study, we have introduced a promising application of hFMSC secretome therapy, and established the positive effect of hFMSC secretome on osteogenic differentiation of rBMSCs and the therapeutic potential to promote bone consolidation in a rat DO model.

MSCs have the ability to differentiate into various specific cell types and promote tissue regeneration both by replacing damaged tissues and by trophic and paracrine mechanisms [6]. Recently, hFMSCs have been demonstrated to be the most promising cell source for bone tissue engineering application because of their lower immunogenicity, and higher proliferative and osteogenic capacity compared to hAMSCs [13, 25]. However, the stem cells have poor differentiation and a poor survival rate following their transplantation in vivo that has limited their regenerative potential [26]. Although the proliferative and differentiation capacities of hFMSCs can be retained and their osteogenic potentials may be enhanced through gene modulation [25, 27], the genetic modification procedure is rather complicated and further studies are still needed before their clinical application. In contrast, cell-free secretome harvested from the hFMSC conditioned medium centrifugation is an easy and cost-effective procedure. To rule out the possible effects of serum factors, we cultured rBMSCs, hFMSCs, and hAMSCs in serum-free medium before we collected the secretome. MSC secretome contains many innate immunomodulatory and trophic factors that will benefit tissue repair [14].

In the current study, we showed that all three kinds of secretome (rBMSCs, hAMSCs, and hFMSCs) promoted osteogenic differentiation of rBMSCs, and the human MSC secretome could act similar to or even better than rat MSC secretome. The hFMSC secretome showed the strongest osteogenic induction ability. Most interestingly, compared to the hAMSC secretome, the hFMSC secretome did not trigger any significant immune response, and hence the potential non-specific response caused by human protein in rats was disregarded in the current study using the hFMSC secretome. Furthermore, hFMSC secretome at different doses did not affect rBMSC viability or cell proliferation, and the concentration of 100 μg/μl could significantly enhance ALP activity and formation of calcium nodules during rBMSC osteogenic induction, indicating enhancement of mineralization [28]. The expression level of some osteogenic marker genes including Runx2, OCN, OPN, and Osx were all significantly upregulated at days 3 and 10 following hFMSC secretome treatment. ALP can promote mineralization through its ability for hydrolyzing pyrophosphate and generating inorganic phosphate, and is responsible for osteoblastic differentiation at an early stage, while OCN plays an important role in the late stage of mineralization [29, 30]. Runx2 is involved in the production of bone matrix proteins and is essential for osteoblast differentiation [31]. Expression levels of OPN can be upregulated by growth and differentiation factors to promote bone formation and remodeling [32]. Osx, downstream of Runx2, is required for osteoblast differentiation and bone formation [33]. Taken together, we confirmed that hFMSC secretome enhanced osteogenic differentiation of rBMSCs in vitro. When we administered hFMSC secretome locally into the DO gap in rats, new bone formation and consolidation were significantly accelerated. This was also confirmed by histological analysis results, including Trichrome Goldner and Von Kossa staining, and the dynamic histomorphometric data, while most of the cartilage tissues in the distraction zone in the PBS group and the medium group remained unconsolidated in the callus. The healing quality of new bone in the distraction zone was quantified by a mechanical test and μCT. E-modulus, ultimate load, energy to failure, and BV/TV all have better results, demonstrating more callus formation and mineralization in the secretome treatment group.

The DO procedure stimulates the recruitment and proliferation of bone progenitor cells to the target site, where they promote angiogenesis and bone formation/mineralization [6, 34]. Mechanisms as to how hFMSC secretome augments bone formation and maintains vascularity are still unclear. Recently, Karp and Leng Teo have demonstrated that the effect of MSC secretome on tissue repair can be improved through enhancing survival of the progenitor cells that homed to the target site [35], whereas the release of vascular endothelial growth factor (VEGF) was activated by MSC secretome under hypoxia or normoxia stimulation [36]. The study of Jacobsen et al. showed that VEGF is highly expressed in the formation of both bone and blood vessels during DO and is needed to promote osteogenic differentiation over chondrogenic differentiation of MSCs [37]. That might be the reason for more cartilage still remaining in the PBS group and medium group in our current study. Moreover, once committed to the osteogenic lineage, MSCs express Osx and OCN which are markers of osteoprogenitors [24, 38]. The number of Osx- and OCN-positive osteoprogenitors was significantly upregulated in the distraction zone after treatment with hFMSC secretome compared to the control treatment groups. A wide array of signaling pathways may be involved in VEGF production following hFMSC secretome treatment, and further research is warranted to verify the current findings.

Despite our promising findings, there are several limitations in the current study. On the one hand, the effectiveness and potential immunogenicity of using human proteins in rats still need further careful investigation. Despite our data confirming that hFMSC secretome had significantly enhanced osteogenic differentiation of rBMSCs and caused no detectable immunological responses, their exact mechanisms of action need further exploration. On the other hand, the microenvironments in vivo are highly dynamic, and the secretome contents likely change from what they would be in vitro; it will be interesting to examine the properties of hFMSC secretome changes over time or at pre- and post-transplantation stages. Meanwhile, future research endeavors should emphasize developing slow-releasing methods to maintain sustainability of the secretome under a dynamically changing microenvironment such as the regenerates of DO.

## Conclusions

In conclusion, our data confirmed that hFMSC secretome improves the osteogenic differentiation potential of rBMSCs and accelerates bone consolidation during DO in a rat model. A novel application of hFMSC secretome is proposed with a clinical potential to enhance bone consolidation of DO treatment for patients with limb discrepancy, severe deformities, and bone defects.

## Abbreviations

3D: Three dimensional
ALP: Alkaline phosphatase
BFR/BS: Bone formation rate per unit of bone surface
BFR/BV: Bone formation rate of bone volume
BFR/TV: Bone formation rate of tissue volume
BV/TV: Bone volume/total tissue volume
CPC: Cetylpyridinium chloride
dL.S: Double-labeled surface
DO: Distraction osteogenesis
E-modulus: The modulus of elasticity
FBS: Fetal bovine serum
GAPDH: Glyceraldehyde-3-phosphate dehydrogenase
hAMSCs: Human adult MSCs
hFMSCs: Human fetal MSCs
MAR: Mineral apposition rate
MS/BS: Ratio of mineralizing surface to bone surface
MSCs: Mesenchymal stem cells
OCN: Osteocalcin
OIM: Osteogenic induction medium
OPN: Osteopontin
Osx: Osterix
PBS: Phosphate-buffered solution
rBMSCs: Rat bone marrow-derived MSCs
Runx2: Runt-related transcription factor 2
SD: Sprague-Dawley
sL.S: Singled labeled surface
VEGF: Vascular endothelial growth factor
α-MEM: Modified Eagle’s medium of Alpha
μCT: Micro-computed tomography
